# Phonemes based detection of parkinson’s disease for telehealth applications

**DOI:** 10.1038/s41598-022-13865-z

**Published:** 2022-06-11

**Authors:** Nemuel D. Pah, Mohammod A. Motin, Dinesh K. Kumar

**Affiliations:** 1 Electrical Engineering Department, Universitas Surabaya, Surabaya, Indonesia; 2 School of Engineering, RMIT University, Melbourne, VIC 3000 Australia; 3 Department of Electrical and Electronic Engineering, Rajshahi University of Engineering and Technology, Rajshahi, Bangladesh

**Keywords:** Neurological disorders, Diagnostic markers, Biomedical engineering, Electrical and electronic engineering

## Abstract

Dysarthria is an early symptom of Parkinson’s disease (PD) which has been proposed for detection and monitoring of the disease with potential for telehealth. However, with inherent differences between voices of different people, computerized analysis have not demonstrated high performance that is consistent for different datasets. The aim of this study was to improve the performance in detecting PD voices and test this with different datasets. This study has investigated the effectiveness of three groups of phoneme parameters, i.e. voice intensity variation, perturbation of glottal vibration, and apparent vocal tract length (VTL) for differentiating people with PD from healthy subjects using two public databases. The parameters were extracted from five sustained phonemes; /a/, /e/, /i/, /o/, and /u/, recorded from 50 PD patients and 50 healthy subjects of PC-GITA dataset. The features were statistically investigated, and then classified using Support Vector Machine (SVM). This was repeated on Viswanathan dataset with smartphone-based recordings of /a/, /o/, and /m/ of 24 PD and 22 age-matched healthy people. VTL parameters gave the highest difference between voices of people with PD and healthy subjects; classification accuracy with the five vowels of PC-GITA dataset was 84.3% while the accuracy for other features was between 54% and 69.2%. The accuracy for Viswanathan’s dataset was 96.0%. This study has demonstrated that VTL obtained from the recording of phonemes using smartphone can accurately identify people with PD. The analysis was fully computerized and automated, and this has the potential for telehealth diagnosis for PD.

## Introduction

Parkinson’s disease (PD) is the second most common neurodegenerative disorder^1^ and its prevalence is expected to increase with an aging population. It is multisymptomatic with a number of motor and non-motor impairments^2,3^. Its diagnosis is based on clinical assessment and the presence of two or more motor symptoms of tremor, rigidity, bradykinesia, or postural impairment or non-motor symptoms such as dysarthria, functional impairment or cognitive impairment are indicative of the disease^4^.

One of the early symptoms of PD is speech impairment, termed as Parkinsonian hypokinetic dysarthria. Speech symptoms are reported by 90% of people with PD^5,6^. The evaluation of Parkinsonian speech reveals a variety of disturbances such as reduced voice intensity, increased voice nasality, increased acoustic noise, reduced speech prosody, imprecise articulation, significantly narrower pitch range, mono loudness, longer pauses, vocal tremor, harsh and breathy voice quality, and disfluency^7,8^. Many of these are based on speech, which are limited by factors such as language skills or poor visual and auditory functions. Voice-based assessments have the advantage that these are more universal^9,10^.

Hypokinetic dysarthria is caused by poor activation and coordination of the speech production muscles^8,11^. The stiffness and tremor of the larynx muscle harden the vocal cords affects the vibration of the vocal cords and causes changes to the fundamental frequency, inadequate closed phases, and irregular or asymmetrical vocal motion during phonation^8,12^. The reduced controllability of the diaphragm muscles causes unstable phonatory airflow and pneumatic pressure to the larynx^8,13,14^. People with PD also have reduced control of other vocal tract muscles such as the tongue and lips.

The standard clinical method for classifying parkinsonian voice is by perceptual evaluation, which however is subjective^15^. Computerized voice analysis has been proposed for a more accurate, objective, and quantifiable alternative, which could also have the potential for telehealth and remote monitoring of the patients.

Studies on the effective Parkinsonian speech and voice biomarkers are clustered into four aspects: phonatory, articulatory, prosodic, and linguistic^16^. The study based on articulatory, prosodic, and linguistic aspects^17^ involves broad factors such as the psychology, linguistics, and cognitive conditions of patients. On the other hand, phonatory aspects of a sustained phoneme are less influenced by the above factors.

Studies have investigated the effectiveness of sustained phoneme parameters in representing the phenomenon of Parkinsonian hypokinetic dysarthria^16,18–21^. Most of the studies were focused on the parameters that are closely related to impairments in vocal cord vibration. The pitch frequency variation, number of pulses, jitter (perturbation of the glottal vibration period), shimmer (amplitude perturbation of glottal vibration), autocorrelation, and harmonics to noise ratio (HNR/NHR) were used in the authors previous work^22^, as well as in the work of Orozco-Arroyave^23^, Behroozi et al.^24^, Tsanas and Little^25^, Ali et al.^26^, Sakar et al.^19^, and Rusz et al.^6^.

Machine-based analysis can be correlated with perceptual features such as voice quality, loudness, pitch, and resonance. Some of the characteristics that have been assessed and found suitable for Parkinsonian voice are vocal intensity, jitter (frequency variability), shimmer (amplitude variability), harmonics to noise ratio (HNR), fundamental frequency (*F*_*0*_), and formant frequency profiles^19,23,25–29^.

Speech production features extracted from the glottal waveform remove the effect of articulation on the acoustic signal. They approximate the volume velocity of the air flowing through the vocal folds and may have an advantage for the analysis of the pathological voice.

Physiologically, these glottic source features are associated with (1) the frequency, amplitude, symmetry, and periodicity of vocal fold vibration; (2) the competency of glottic closure, and (3) speed of the vibratory cycle and the ratio of its open to closed phases. Breathiness, the hallmark perceptual voice quality of parkinsonian speech, is associated with incomplete closure of the vocal folds leading to air escape, and thus the presence of relatively higher noise in the voice, lowered the intensity and a predominance of the open phase of glottic pulse^8,30^. People with PD have higher jitter and lower HNR, associated with aperiodicity of vocal fold vibration and perceived as roughness. Connected speech of people with PD is monotonous and has reduced pitch and loudness variation.

Perez^31^ combined the above parameters with thirteen Mel Frequency Cepstral Coefficients (MFCCs) that represent the energy and articulatory positions. Fractal dimension (FD) features that measure the complexity of the signal was used by Viswanathan et al.^32^. More recently, multivariate deep-features have been found to be effective^33^.

Even though the above studies have demonstrated some significant differences between the voice parameters of controls and people with PD, their implementation in a generalized automatic system is not straightforward^34^. There is also evidence of inconsistent results between different studies^32^.

Gillivan-Murphy^35^ published preliminary findings based on nasolaryngoscopy which shows that PD voice tremor is not associated with the vocal folds. PD voice tremor is likely to be related to oscillatory movement in structures across the vocal tract rather than just the vocal folds. Furthermore, pronouncing a phoneme is a voluntary activity while PD tremors exist during rest. This may result in an inconsistent appearance of voice tremor in sustained and steady phoneme recordings which is essential for glottal vibration parameters.

The parameters other than the glottal vibration parameters that may potentially be used in PD identification are the parameters related to phonatory airflow and pneumatic pressure to the larynx such as voice intensity and the parameters related to vocal tract muscles such as formants and Vocal Tract Length (VTL)^36,37^.

This study has investigated and compared the effectiveness of three groups of parameters to differentiate the voice of people with PD from that of age-matched healthy participants. These are related to three domains of speech production control: (i) the stability of lung control, (ii) the periodicity and stability of glottal vibration control, and (iii) the stability of vocal tract control. Standard deviation (SD) and range of phonemes intensity were used to measure the lung stability while the shimmer, jitter, SD of pitch, and harmonics parameters were used for the stability of glottal vibration. The vocal tract stability was represented by the SD of the first four formants and the apparent Vocal Tract Length (VTL).

The comparison was examined using a statistical hypothesis test, followed by classification using the Support Vector Machine (SVM). The parameters were extracted from the recordings of sustained phonemes /a/, /e/, /i/, /o/, and /u/. Public database PC-GITA was used for this study. To evaluate the consistency of the method between different datasets, the SVM classifications were also applied to Viswanathan’s dataset^38^ which contains the recordings of /a/, /o/, and /m/.

## Methods

### Database of recordings

Two databases of recordings were used in this study. The first is the publicly available database, PC-GITA, provided by Rafael Orozco et al.^23^. It contains the recordings of 100 Columbian-Spanish native speakers, 50 of them were diagnosed with PD, and the other 50 were age and gender-matched participants with no PD or any other neurological disease symptoms. Table 1 presents participants’ demographic and clinical information. The *p-values* in the table confirm that there was no significant age difference between the groups as well as showing the matched clinical stage between male and female groups of PD subjects. The speech recording of the PD subjects was conducted within 3-h after their morning medication and hence has been in pharmacological ON-state. The procedure complied with the Helsinki Declaration and was approved by the Ethics Committee of the Clinica Noel, in Medellin, Colombia.

**Table 1:** Participants’ demographics of PC-GITA database.

	PD subjects		Control subjects		p-value
	Male	Female	Male	Female	
# Subjects	25	25	25	25	
Age (years)	61.56 ± 11.63	60.72 ± 72.66	60.36 ± 11.56	61.44 ± 6.98	0.966*
UPDRS	35.92 ± 22.77	37.56 ± 14.03			0.760^+^
H&Y	2.30 ± 0.94	2.28 ± 0.54			0.927^+^
Years diagnosed	8.86 ± 5.88	12.58 ± 11.52			0.157^+^

*Calculated using ANOVA with 95% confidence level.

^+^Calculated using unpaired T-test with 95% confidence level.

**Figure 1:**
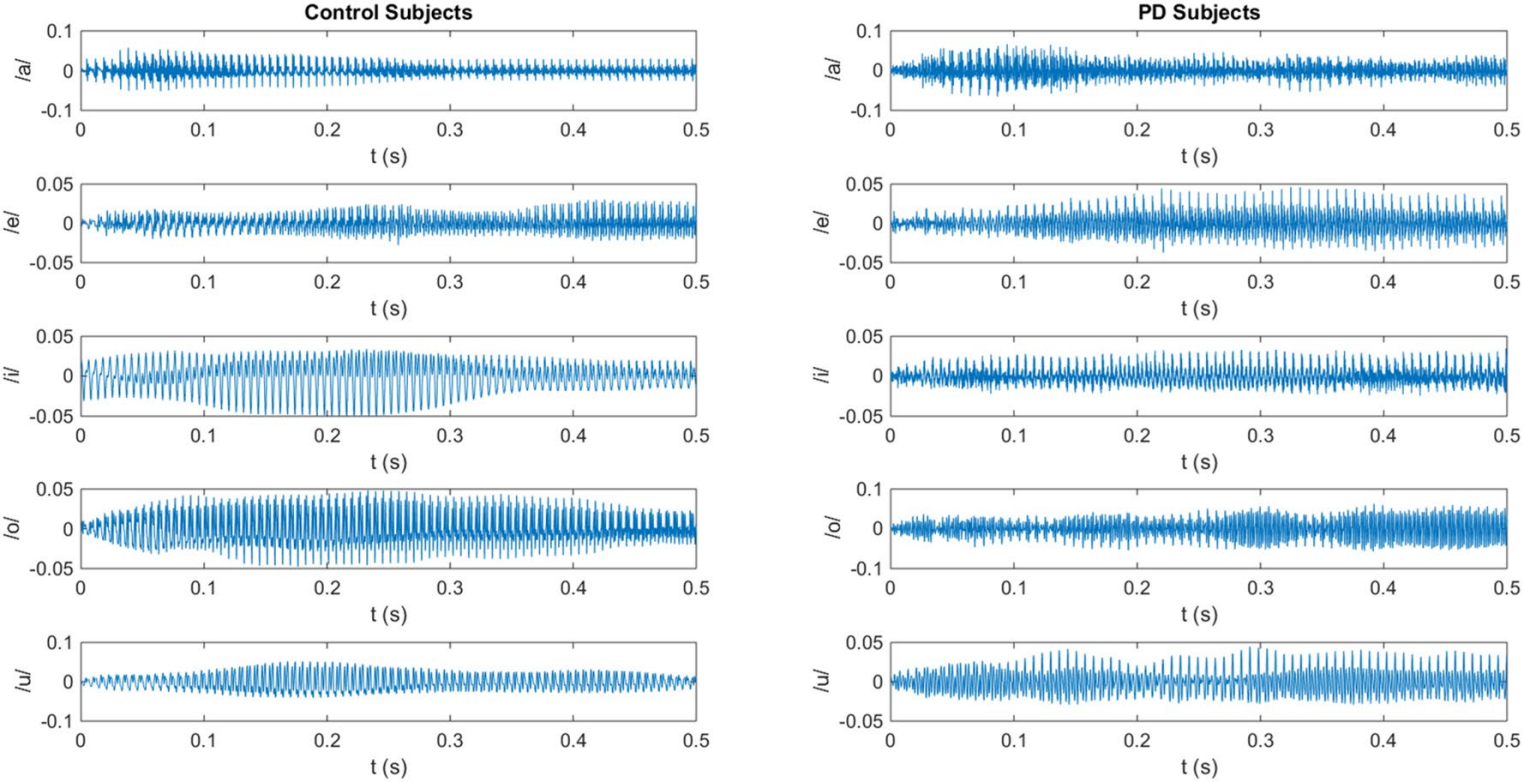
The waveforms of the five vowels recorded from the control subjects and the PD subjects.

The recordings were captured in noise-controlled conditions and sampled at 44,100 Hz with 16 resolution bits, using a dynamic omnidirectional microphone (Shure, SM 63L). In this study, we use the recording of the five vowels /a/, /e/, /i/, /o/, and /u/. The participants produced three repetitions of the sustained vowel, each done as long as possible in one breath, at their natural pitch and loudness. Figure 1 illustrates the waveforms of the five vowels recorded from control and PD patients.

The second is the Viswanathan’s dataset^32^ available publicly on request. This has the recordings from 24 people with PD and 22 people with no neurological disease and age-matched with PD, referred to as Controls. The people with PD were recruited from the Movement Disorders Clinic at Monash Medical Centre, Australia. All people with PD have been diagnosed within the last ten years. Three sustained phonemes /a/, /o/, and /m/ were recorded from each participant in a noise-restricted environment using Samson-SE50 microphone. The recordings were stored in a single-channel WAV format with a sampling rate of 48 kHz and a 16-bit resolution. The sustained phonemes of people with PD in the database were recorded in *on*-state and *off*-state medication. However, for this study, only the *on*-state recordings were used. Table 2 provides the demographics of the subjects. The detailed information can be found in^22,32^.

**Table 2:** Participants’ demographics of Viswanathan’s database.

	Control Subjects	PD Subjects	p-value
Number of subjects	22	24	
Age	66.30 ± 6.20	71.92 ± 7.07	0.008
PD-*off* MDS-UPDRS-III score	N/A	25.54 ± 8.78	1.42e−05 (PD-off vs PD-on)
PD-*on* MDS-UPDRS-III score	N/A	19.33 ± 9.30	
MoCA	28.30 ± 1.34	27.25 ± 2.67	0.118
Duration of disease (years)	N/A	5.29 ± 2.99	

### Parameter extraction

A publicly available speech analysis software, Praat^39^, was used to extract speech features from the recordings. Before features extraction, the recordings were trimmed to a uniform duration of 0.5 s based on the assumption that vowels correspond to largely stationary signals. The recordings were filtered with an IIR 4th order Butterworth band-pass filter of 50 Hz to 4 kHz.

#### Voice intensity parameters

The voice intensity is controlled by the subglottal pressure, which is controlled by the respiratory muscles and the lung volume^40^ and thus, it is hypothesized that people with PD will have increased variation and reduced range of the voice intensity. The standard deviation and range of intensity are proportional to the fluctuation of lung pressure during the pronunciation of the sustained phoneme that may capture the tremor or rigidity due to Parkinson's disease.

The standard deviation and range of voice intensity were obtained for each recording. The parameters measure the ability of the subject to keep the stability of air pressure produced by the lung. The intensity, *I* (in dB), of an input voice *s(t)* with a duration of *T,* were calculated using Praat’s function with energy averaging method as in Eq. (1).

1 $$I=10 {log}_{10}\frac{1}{T}{\int }_{0}^{T}{10}^{\frac{s(t)/}{10}}dt$$

#### Periodicity and stability of glottal vibration

It is commonly assumed that Parkinsonian dysarthria is affected by the abnormal vibration of the vocal cords, such as the inadequate or excessive closing of the vocal cords and irregular or asymmetrical vocal fold, as well as a tremor in its muscles^8,34,35^. A total of 6 parameters related to the periodicity and stability of glottal vibration were extracted from each recording. The parameters were jitter absolute (*abs*), jitter relative (*rel*), the absolute shimmer (in dB), the relative shimmer, the standard deviation of pitch frequency (*f*_*0*_), the *HNR,* and the *NHR.*

The jitter parameters^41^ were related to time perturbation glottal pulses, *T*_*i*_. The equation to calculate the two jitter parameters^41^ are shown in Eqs. (2) and (3):

2 $$Jitter\left(abs\right)=\frac{1}{N-1}\sum_{i=1}^{N-1}\left|{T}_{i+1}-{T}_{i}\right|$$

3 $$Jitter\left(rel\right)=\frac{\frac{1}{N-1}\sum_{i=1}^{N-1}\left|{T}_{i+1}-{T}_{i}\right|}{\frac{1}{N}\sum_{i=1}^{N}{T}_{i}}$$

The shimmer parameters^41^ were related to amplitude perturbation of the glottal cycles. The parameters were calculated with Eqs. (4) and (5):

4 $$Shimmer\left(abs,dB\right)=\frac{1}{N-1}\sum_{i=1}^{N-1}\left|20*\mathrm{log}\left(\frac{{A}_{i+1}}{{A}_{i}}\right)\right|$$

5 $$Shimmer\left(rel\right)=\frac{\frac{1}{N-1}\sum_{i=1}^{N-1}\left|{A}_{i+1}-{A}_{i}\right|}{\frac{1}{N}\sum_{i=1}^{N}{A}_{i}}$$

The standard deviation of the pitch was calculated based on the instantaneous pitch frequency *f*_*0 i*_ = *1/T*_*i*_. The HNR and NHR were calculated based on the normalized autocorrelation function of the segment. *R*_*xx*_*[T*_*0*_*]* is the peak next to the center of *R*_*xx*_ at a distance corresponding to the *T*_*0*_ of the recording. The HNR and NHR were calculated as described in Eqs. (6) and (7)^42,43^:

6 $$HNR=10*log\frac{{R}_{xx}[{T}_{0}]}{1-{R}_{xx}[{T}_{0}]}$$

7 $$NHR=1-{R}_{xx}[{T}_{0}]$$

##### Formants parameters

The limitations of the control in the speech production process by the people with PD leads to some disturbances including the change in phonatory and resonant characteristics^34^. The disturbances in the resonant characteristics are due to an inaccurate position of the articulators or a lack of control of vocal tract muscles. The accurate position and control of vocal tract muscles can be observed in the fluctuation of formants frequencies. The stability of vocal tract control in this study was measured with a standard deviation of the first four formants (*F*_*1*_, *F*_*2*_, *F*_*3*_, and *F*_*4*_) and the Vocal Tract Length (VTL). The formants of each recording were extracted from Praat using Burg’s method^44^ with a maximum formant value of 5.5 kHz, a window length of 25 ms, a time step of 6.25 ms, and a pre-emphasis from 50 Hz. The mean and standard deviation were then calculated for each recording.

##### Vocal tract length

The other parameter that captures the resonant characteristic of the vocal tube model of voice production is the apparent vocal tract length (VTL). VTL is the estimation of the physical vocal tract length of a subject while pronouncing a specific voice based on formants frequency. VTL has been used in other voice analyses such as speaker verification^45^, identifying body measures^36,46^.

VTL of each recording was calculated (in cm) from the mean of the four formants, *F*_*i*_, with the formula in Pisanski et al.^36^.

8 $$VTL({F}_{i})=(2i-1)\frac{c}{4 {F}_{i}}$$

The constant, *c* = 33,500 cm/s, is the speed of sound in a uniform tube with one end closed. A total of four VTL were calculated for each recording associated with each formant, *F*_*i*_.

### Statistical analysis

The mean and standard deviation of all the parameters were computed for the two groups of the PC-GITA database: PD and CO. The normality of the extracted parameters was examined with the Anderson–Darling test^47^. Mann Whitney U-test^48^ was used to compare the group differences for speech parameters between PD and control subjects. The 95% confidence level was considered for the analysis and p-value < 0.05 to indicate that the mean of the groups was significantly different. All the statistical analyses were performed using MATLAB2018b (MathWorks).

### Support vector machine classification

The effectiveness of the parameters to classify PD and control subjects was investigated with Support Vector Machines (SVM)^49^ classifier. The SVM was trained with a Gaussian kernel and validated using “leave-one-out” cross-validation. The Gaussian kernel was selected anecdotally since it yielded the best result compared to the other kernels. The input to the SVM were the sets of voice parameters and the ten highest-ranked features, selected using the Relief-F algorithm^50^ with 10 nearest neighbors (*k* = 10). The classification accuracy, sensitivity, and selectivity were evaluated based on the true-positive (TP), true-negative (TN), false-positive (FP), and false-negative (FN).

### Ethics

This paper reports the analysis of two datasets: Viswanathan and PC-GITA. Viswanathan dataset was developed using the research protocol for analysis was approved by RMIT University human experiments Committee for Ethics in Human Research and the experiments were performed in accordance with Helsinki declaration for ethical experiments, revised 2013. PC-GITA dataset was developed based on the procedure that complied with the Helsinki Declaration and was approved by the Ethics Committee of the Clinica Noel, in Medellin, Colombia. Both database confirm that all participants provided written consent for the experiments.

## Results

### Statistical analysis

The Anderson–Darling test confirmed that except for some VTL parameters, the parameters were not normally distributed. Mann Whitney U-test, a non-parametric test, was thus used to test for group differences in each of the features. Table 3 provides the statistical distribution (mean ± SD) and *p-value* and effective size of Mann Whitney U-test between CO and PD for all the features. The table shows that the parameters of people with PD fluctuated more than CO. The voice intensity of people with PD has both higher SD and range, which indicates their diminished ability to produce sustained phonemes with stable air pressure. The p < 0.05 shows that the group difference was significant.

**Table 3:** Statistical distribution and the result of Mann Whitney U-test.

Parameters	Phoneme	Mean ± SD		p-value	Effect Size	Parameters	Phoneme	Mean ± SD		p-value	Effect size
		Control	PD					Control	PD		
Intensity (SD)	a	1.62 ± 0.85	1.95 ± 1.25	0.051	− 0.387	F_1_(SD)	a	4.87E+1 ± 4.93E+1	7.41E+1 ± 8.22E+1	0.000	− 0.515
	e	1.66 ± 1.03	2.21 ± 1.35	0.000	− 0.528		e	2.87E+1 ± 5.03E+1	3.20E+1 ± 3.66E+1	0.001	− 0.065
	i	1.84 ± 1.03	2.25 ± 1.36	0.018	− 0.394		i	4.08E+1 ± 1.16E+2	5.48E+1 ± 1.26E+2	0.001	− 0.120
	o	1.79 ± 1.01	2.15 ± 1.26	0.020	− 0.353		o	4.29E+1 ± 3.57E+1	4.87E+1 ± 3.63E+1	0.018	− 0.163
	u	1.69 ± 1.05	2.35 ± 1.40	0.000	− 0.633		u	4.83E+1 ± 3.92E+1	5.13E+1 ± 4.23E+1	0.412	− 0.078
Intensity (range)	a	6.12 ± 3.11	7.34 ± 4.37	0.039	− 0.392	F_2_(SD)	a	7.89E+1 ± 9.20E+1	1.16E+2 ± 1.44E+2	0.005	− 0.403
	e	6.28 ± 3.63	8.09 ± 4.50	0.000	− 0.498		e	5.73E+1 ± 5.03E+1	7.73E+1 ± 6.75E+1	0.000	− 0.397
	i	6.62 ± 3.37	8.22 ± 4.56	0.004	− 0.473		i	6.93E+1 ± 8.55E+1	1.04E+2 ± 1.21E+2	0.000	− 0.411
	o	6.70 ± 3.65	7.95 ± 4.33	0.010	− 0.343		o	1.77E+2 ± 2.76E+2	1.84E+2 ± 2.50E+2	0.002	− 0.023
	u	6.28 ± 3.45	8.41 ± 4.60	0.000	− 0.615		u	2.91E+2 ± 3.24E+2	2.63E+2 ± 2.98E+2	0.789	0.086
Jitter (abs)	a	4.09E−5 ± 3.32E−5	5.70E−5 ± 5.10E−5	0.005	− 0.485	F_3_(SD)	a	1.09E+2 ± 1.18E+2	1.35E+2 ± 1.22E+2	0.029	− 0.221
	e	3.56E−5 ± 2.96E−5	4.81E−5 ± 3.93E−5	0.001	− 0.424		e	8.97E+1 ± 7.35E+1	1.14E+2 ± 9.06E+1	0.002	− 0.325
	i	3.54E−5 ± 2.98E−5	4.46E−5 ± 4.20E−5	0.037	− 0.308		i	1.12E+2 ± 7.80E+1	1.38E+2 ± 9.85E+1	0.017	− 0.334
	o	3.70E−5 ± 3.83E−5	4.82E−5 ± 5.30E−5	0.100	− 0.292		o	1.21E+2 ± 1.38E+2	1.20E+2 ± 1.04E+2	0.030	0.008
	u	2.90E−5 ± 2.02E−5	4.35E−5 ± 4.31E−5	0.005	− 0.716		u	1.87E+2 ± 1.74E+2	1.80E+2 ± 1.68E+2	0.812	0.041
Jitter (rel)	a	5.84E−3 ± 3.55E−3	8.62E−3 ± 7.11E−3	0.002	− 0.782	F_4_(SD)	a	1.66E+2 ± 1.48E+2	1.84E+2 ± 1.47E+2	0.308	− 0.119
	e	5.07E−3 ± 3.21E−3	7.69E−3 ± 6.28E−3	0.000	− 0.815		e	1.91E+2 ± 1.72E+2	1.73E+2 ± 1.50E+2	0.581	0.105
	i	5.27E−3 ± 3.18E−3	7.31E−3 ± 6.51E−3	0.006	− 0.641		i	1.71E+2 ± 1.59E+2	1.61E+2 ± 1.32E+2	0.828	0.064
	o	5.30E−3 ± 4.21E−3	7.53E−3 ± 7.68E−3	0.011	− 0.530		o	1.64E+2 ± 1.69E+2	1.60E+2 ± 1.30E+2	0.194	0.027
	u	4.60E−3 ± 2.43E−3	7.25E−3 ± 6.46E−3	0.000	− 1.094		u	2.37E+2 ± 2.13E+2	2.07E+2 ± 1.66E+2	0.733	0.143
Shimmer (abs)	a	4.72E−1 ± 2.33E−1	6.11E−1 ± 3.07E−1	0.000	− 0.596	VTL(F_1_)	a	10.73 ± 1.75	11.11 ± 1.88	0.101	− 0.216
	e	4.45E−1 ± 2.07E−1	5.77E−1 ± 2.71E−1	0.000	− 0.638		e	17.59 ± 2.43	17.97 ± 2.48	0.291	− 0.157
	i	4.30E−1 ± 2.06E−1	5.19E−1 ± 2.43E−1	0.000	− 0.436		i	23.66 ± 4.68	23.98 ± 4.56	0.436	− 0.068
	o	4.02E−1 ± 1.84E−1	5.45E−1 ± 3.20E−1	0.000	− 0.772		o	16.27 ± 2.20	16.57 ± 2.75	0.447	− 0.140
	u	3.86E−1 ± 2.05E−1	5.24E−1 ± 2.87E−1	0.000	− 0.671		u	19.92 ± 3.28	20.90 ± 4.02	0.090	− 0.300
Shimmer (rel)	a	4.93E−2 ± 2.55E−2	6.47E−2 ± 3.44E−2	0.000	− 0.606	VTL(F_2_)	a	18.82 ± 2.40	18.01 ± 2.77	0.001	0.337
	e	4.58E−2 ± 2.21E−2	5.91E−2 ± 3.03E−2	0.000	− 0.601		e	12.04 ± 1.22	12.05 ± 1.55	0.817	− 0.005
	i	4.39E−2 ± 2.20E−2	5.28E−2 ± 2.74E−2	0.001	− 0.407		i	10.80 ± 1.05	11.15 ± 1.43	0.088	− 0.336
	o	4.03E−2 ± 1.95E−2	5.51E−2 ± 3.60E−2	0.000	− 0.759		o	26.76 ± 5.18	25.42 ± 5.33	0.030	0.258
	u	3.88E−2 ± 2.06E−2	5.26E−2 ± 3.28E−2	0.000	− 0.672		u	27.25 ± 8.78	25.91 ± 8.55	0.059	0.153
Pitch(SD)	a	8.56E+0 ± 9.51E+0	1.30E+1 ± 1.58E+1	0.076	− 0.470	VTL(F_3_)	a	15.89 ± 1.27	15.51 ± 1.43	0.024	0.293
	e	8.59E+0 ± 1.38E+1	1.67E+1 ± 2.39E+1	0.000	− 0.590		e	15.74 ± 1.23	15.48 ± 1.37	0.043	0.209
	i	8.54E+0 ± 1.10E+1	1.24E+1 ± 1.49E+1	0.045	− 0.346		i	14.56 ± 1.13	14.22 ± 1.16	0.002	0.299
	o	1.07E+1 ± 1.48E+1	1.59E+1 ± 2.30E+1	0.035	− 0.350		o	15.45 ± 1.43	15.29 ± 1.62	0.376	0.117
	u	8.74E+0 ± 1.09E+1	1.29E+1 ± 1.52E+1	0.003	− 0.386		u	14.91 ± 1.45	15.06 ± 1.61	0.430	− 0.102
HNR	a	1.89E+1 ± 4.01E+0	1.64E+1 ± 4.77E+0	0.000	0.643	VTL(F_4_)	a	16.08 ± 1.45	16.15 ± 1.30	0.379	− 0.053
	e	1.98E+1 ± 3.96E+0	1.78E+1 ± 4.73E+0	0.000	0.493		e	16.07 ± 1.59	15.93 ± 1.48	0.535	0.085
	i	2.10E+1 ± 4.23E+0	1.99E+1 ± 4.71E+0	0.070	0.257		i	15.76 ± 1.47	15.46 ± 1.51	0.017	0.205
	o	2.41E+1 ± 4.19E+0	2.18E+1 ± 5.33E+0	0.000	0.553		o	15.95 ± 1.27	15.74 ± 1.28	0.128	0.163
	u	2.62E+1 ± 4.42E+0	2.39E+1 ± 5.04E+0	0.000	0.524		u	15.24 ± 1.30	15.16 ± 1.45	0.467	0.062
NHR	a	5.11E−2 ± 4.32E−2	8.66E−2 ± 8.37E−2	0.000	− 0.823						
	e	3.69E−2 ± 3.00E−2	6.50E−2 ± 6.83E−2	0.000	− 0.937						
	i	3.12E−2 ± 2.92E−2	4.53E−2 ± 4.55E−2	0.007	− 0.486						
	o	3.02E−2 ± 3.19E−2	4.74E−2 ± 6.60E−2	0.015	− 0.541						
	u	1.80E−2 ± 1.97E−2	3.17E−2 ± 4.56E−2	0.002	− 0.697						

The statistical distribution of the glottal vibration parameters, i.e., jitter, shimmer, SD of pitch, was significantly higher for people with PD compared to the CO, with p-value < 0.05. The HNR and NHR distribution show that PD voice had higher noise (non-periodic) components compared to healthy people.

For vocal tract parameters, except for phoneme /o/ and /u/, the first three formants (*F*_*1*_, *F*_*2*_, and *F*_*3*_) of PD patients have a significantly higher standard deviation compared to the normal subjects. The majority of VTL parameters did not show significant differences between PD and normal subjects. The p-value and effect size confirm that statistically, the mean of the groups was not significantly different.

### SVM classification

The SVM classification results of recordings from the PC-GITA database for the four groups of input parameters are shown in Table 4. It presents the accuracy, sensitivity, and selectivity when considering each vowel independently and with the combination of the five vowels. For the sake of presentation simplicity and without loss to the outcome of this work, the table only presents the results of the vowel combination with significant accuracy. The results show that the classification accuracy of 84.3% was obtained with the combination of all the vowels when the SVM input were VTL(F_i_); the overall observation is that VTL is the most effective feature to distinguish between voice of PD and CO. The SVM classification accuracy was 71.2% when it was given the ten highest-ranked features selected by the Relief-F algorithm. The ten highest-ranked features selected by Relief-F algorithm were dominated by the VTL (VTL(F4) of/o/; VTL(F1) of /i/;VTL(F2) of /o/; VTL(F3) of /u/; std(F1) of /o/; std(F2) of /o/; VTL(F1) of /e/; VTL(F1) of /a/; VTL(F2) of /i/; VTL(F2) of /u/). Comparing the vowels, the VTL of /i/ was the most effective parameter with an accuracy of 73.0%. The percentage of sensitivity and selectivity was about at the same level as the accuracy for almost all the input configurations.

**Table 4:** The SVM classification results of PC-GITA database.

Input parameter to SVM	Phoneme	Accuracy (%)	Sensitivity (%)	Specificity (%)
[SD(Intensity), range(Intensity)]	/a/	53.3	56.7	50.0
	/e/	49.7	60.0	39.3
	/i/	56.7	64.0	49.3
	/o/	48.3	58.0	38.7
	/u/	50.3	54.7	46.0
	/e/ + /o/ + /u/	**77.3**	**81.3**	**73.3**
[Jitt(abs), Jitt(rel), Shim(abs), Shim(rel), SD(pitch), HNR, NHR]	/a/	59.9	64.4	55.3
	/e/	61.5	63.1	60.0
	/i/	65.2	69.8	60.7
	/o/	61.2	63.8	58.7
	/u/	62.5	72.5	52.7
	/e/ + /i/ + /o/	**70.9**	**74.5**	**67.3**
[SD(F_1_), SD(F_2_), SD(F_3_), SD(F_4_)]	/a/	61.3	72.7	50.0
	/e/	62.0	71.3	52.7
	/i/	59.0	70.7	47.3
	/o/	57.3	72.0	42.7
	/u/	54.3	46.7	62.0
	/a/ + /e/ + /i/ + /o/ + /u/	**68.0**	**72.7**	**63.3**
[VTL(F_1_), VTL(F_2_), VTL(F_3_), VTL(F_4_)]	/a/	69.3	70.7	68.0
	/e/	65.3	64.7	66.0
	/i/	73.0	76.0	70.0
	/o/	66.3	70.7	62.0
	/u/	63.7	67.3	60.0
	/a/ + /e/ + /i/ + /o/ + /u/	**84.3**	**84.0**	**84.7**
Ten highest-ranked features selected by Relief-F: VTL(F4) of /o/; VTL(F1) of /i/; VTL(F2) of /o/; VTL(F3) of /u/; std(F1) of /o/; std(F2) of /o/; VTL(F1) of /e/; VTL(F1) of /a/; VTL(F2) of /i/; VTL(F2) of /u/		**71.2**	**70.5**	**72.0**

Significant values are in bold.

To evaluate the consistency of SVM classification using VTL(F_i_) in different databases, the SVM classifications using VTL(F_i_) were also applied to Viswanathan’s dataset^38^ which contains the recordings of /a/, /o/, and /m/. Table 5 provides the classification results of the recordings in the database. The table shows that the SVM classification using VTL(Fi) as input parameters performs consistently with different databases. The highest accuracy was 96.0% with the combination of VTL(Fi) of /a/ and /m/, while an accuracy of 94.0% was obtained with the combination of /a/, /o/, and /m/.

**Table 5:** The SVM classification results of Viswanathan’s database.

Input parameter to SVM	Phoneme	Accuracy (%)	Sensitivity (%)	Specificity (%)
[VTL(F_1_), VTL(F_2_), VTL(F_3_), VTL(F_4_)]	/a/	85.8	87.0	84.5
	/o/	79.0	77.5	80.5
	/m/	87.8	87.5	88.0
	/a/ + /o/	90.0	90.5	89.5
	**/a/ + /m/**	**96.0**	**96.5**	**95.5**
	/o/ + /m/	91.3	91.0	91.5
	/a/ + /o/ + /m/	94.0	93.5	94.5

Significant values are in bold.

## Discussion

Several earlier studies that have proposed the use of voice-based diagnosis and assessment of Parkinson's disease^16,18–22^. These studies used the vocal cord vibration parameters such as pitch frequency variation, number of pulses, jitter, shimmer, autocorrelation, and harmonics to noise ratio (HNR/NHR). While these studies showed the potential of voice-based biomarkers for Parkinson’s disease, these show inconsistent results in different databases^6,23^. As an example, the vocal cord vibration parameters based analysis gave classification accuracy of 78.1% in Viswanathan’s dataset^22^ but performed poorly for PC-GITA dataset as shown in Table 4 (70.9% of accuracy).

This study has identified VTL as a potential parameter to be used in the classification of PD patients based on sustained phoneme recordings. The parameters have achieved *84.3%* accuracy, *84.0%* sensitivity, *84.7%* specificity when used in PC-GITA database with five vowels /a/, /e/, /i/, /o/, and /u/. This study showed the consistency of the parameters when applied in different datasets. Table 5 shows that when applied in Viswanathan's datasets, VTL parameters could classify PD patients from healthy subjects with an accuracy of 96.0%.

This study has shown that among the features reported in the literature, VTL features are most suitable for differentiating the voice of people with PD from that of Control. VTL is an approximate measure of the physical vocal tract length while producing voice. The shape and length of the vocal tract affect the value and space of formants. Longer vocal tracts produce lower, more closely spaced formants^36^. Although the length of the vocal tract mainly depends on the physical body structure, the study of Piransky et al.^37^ found that a person may voluntarily modify the length of the vocal tract up to 25%. The result reported in this paper indicates the possible relation between the modification of vocal tract length by a subject with a symptom of PD. When a PD patient, due to the reduction in the ability to control speech muscle, modifies the length of the vocal tract, the properties of voice modulation in the vocal tract change. The relation is a higher-order relation. The linear separation by statistic test could not properly separate the PD from healthy subjects.

The novelty of this study is the high performance in differentiating between voices of PD from Controls, and which is consistent for two different databases. We are the first study that investigated the use of VTL to identify voices of people with PD and found that VTL parameters outperformed the features reported in the literature that are related to perturbation of glottal vibration, such as jitter, shimmer, pitch frequency, and harmonics ratio. The finding in this study suggests and supports the argument in^35^ that the neuro-physiology change in PD patients is manifested more in the change of vocal tract control compared to glottal vibration or air pressure control by the lung. This opens the potential for computerized and remote monitoring of people with PD.

The limitation of this study is we have only investigated two databases; Columbian-Spanish native speakers and Australian native speakers. Further study needs to be conducted of people from other demographics and ethnicity to validate the findings for global use. While the size of the datasets are sufficient, larger datasets are required that will allow the examination of the various confounding factors. There is also the need to investigate the effect of PD medication such as Levodopa on these parameters and to test this over repeated voice recordings.

## Conclusion

This study has investigated the effectiveness of using three sets of voice features of sustained phonemes to differentiate people with PD from age-matched healthy participants using two independent and different sets of publicly available databases. It has found that the most effective feature set was using apparent vocal tract length (VTL). The classification accuracy in identifying PD from control was 84.3% when combining the VTL features of all the five vowels /a/, /e/, /i/, /o/, and /u/. The classification accuracy when using /a/, /o/ and /m/ using Viswanathan dataset obtained using smartphone was 96%. This performance was significantly higher than the accuracy obtained when using the glottal vibration parameters (jitter, shimmer, pitch, and harmonics) and voice intensity. Another advantage of VTL parameters is that there were obtained automatically and thus suitable for computerized analysis of the voice recordings using smartphones. Unlike deep-learning approach, this method has the benefit because it has identified the specific voice parameters which allows the clinician to understand the differences. This has the potential for telephone-based diagnosis for PD**.**

## Data Availability

We have used publicly available datasets. GITA dataset is available on request from Orzoco et al. (reference^23^). Viswanathan dataset is available from contact of reference^32^.
